# EW-7197, transforming growth factor β inhibitor, combined with irreversible electroporation for improving skin wound in a rat excisional model

**DOI:** 10.1038/s41598-024-61003-8

**Published:** 2024-06-04

**Authors:** Chu Hui Zeng, Jeon Min Kang, Song Hee Kim, Yubeen Park, Soyeon Shim, Dae-Kee Kim, Ji Hoon Shin, Jung-Hoon Park

**Affiliations:** 1 Biomedical Engineering Research Center, Asan Institute for Life Sciences, Asan Medical Center, 88 Olympic-ro 43-gil, Songpa-gu, Seoul, 05505 Republic of Korea; 2 Department of Radiology and Research Institute of Radiology, Asan Medical Center, University of Ulsan College of Medicine, 88 Olympic-ro 43-gil, Songpa-gu, Seoul, 05505 Republic of Korea; 3 EWHA DrugDesignHouse, 52 Ewhayeodae-gil, Seodaemun-gu, Seoul, 03760 Republic of Korea; 4 Department of Convergence Medicine, Asan Medical Center, University of Ulsan College of Medicine, 88 Olympic-ro 43-gil, Songpa-gu, Seoul, 05505 Republic of Korea

**Keywords:** Diseases, Biotechnology

## Abstract

To evaluate the safety and efficacy of combining EW-7197 with irreversible electroporation (IRE) for improving wound healing, 16 male Sprague–Dawley rats were randomly divided into four groups of four rats each after dorsal excisional wound induction: sham control group; oral administration of EW-7197 for 7 days group; one-time application of IRE group; and one-time application of IRE followed by oral administration of EW-7197 for 7 days group. Measurement of wound closure rate, laser Doppler scanning, histological staining (hematoxylin and eosin and Masson’s trichrome), and immunohistochemical analyses (Ki-67 and α-SMA) were performed to evaluate the efficacy. Fifteen of 16 rats survived throughout the study. Statistically significant differences in wound closure rates were observed between the combination therapy group and the other three groups (all *P* < 0.05). The degrees of inflammation, α-SMA, and Ki-67 were reduced in the EW-7197 and IRE monotherapy groups; however, not statistically significant. The fibrosis score exhibited significant reduction in all three treatment groups, with the most prominent being in the combination therapy group. This study concludes that oral administration of EW-7197 combined with IRE demonstrated effectiveness in improving skin wound in a rat excisional model and may serve as a potential alternative for promoting healing outcomes.

## Introduction

Suboptimal wound healing, resulting from incidents such as trauma and surgery, may form disfiguring scarring and dysfunctional tissue, leading to physical, aesthetic, functional, psychological, and social burdens for affected patients^1,2^. Although scarring typically presents as asymptomatic cosmetic deformities, some may grow to a size that leads to functional limitations, especially when located along the joint^3^. Various therapeutic approaches have been designed to target different stages of wound healing to prevent scar formation, such as surgical excision, intralesional steroid/interferon injection, cryotherapy, laser therapy, silicone sheet application, and combinations thereof^4–6^; however, each technique carries the risk of recurrence, and the improvements in the healing outcomes remain only modest. Since the wound healing process can be easily interfered with, improving wound healing remains a major clinical challenge^7^.

Fibrosis is a pathological feature of wound healing and is characterized by excessive extracellular matrix (ECM) deposition^3^; as a result, attenuating fibrosis is considered a measure to improve wound healing with great potential^2^. In addition to excessive ECM deposition, characteristics of wound healing include several elevated cytokines with minimal anti-inflammatory cytokines and a high concentration of transforming growth factor β (TGF-β). As an important cytokine responsible for excessive scar tissue formation following injuries, the role of TGF-β in wound healing has gained significant attention. The TGF-β/Smad signaling pathway is recognized as a central mediator of wound healing and scarring processes because it regulates the expression of α-SMA and promotes the deposition of collagen I and III at the wound site. Among its three isoforms (TGF-β1, TGF-β2, and TGF-β3), TGF-β1 is the most prevalent and biologically relevant one that is linked to excessive scarring and fibrosis^8^. Therefore, growing evidence supports the notion that blocking TGF-β1, and consequently its effects, can drive the treatment of fibrotic conditions and scarring forward^9^. EW-7197 (2-Fluoro-*N*-[[5-(6-methylpyridin-2-yl)-4-([1,2,4]triazolo[1,5-*a*]pyridin-6-yl)-1*H*-imidazol-2-yl]methyl]aniline; vactosertib), a type I receptor kinase inhibitor of TGF-β, has been introduced as an antifibrotic/cancer immunotherapeutic agent^10^. Featuring a highly potent, selective, and orally bioavailable ALK-5 inhibitor, EW-7197 has previously demonstrated its effectiveness in reducing the formation of peritoneal adhesion, a type of scar tissue connecting opposing organs or the inner abdominal wall after surgery. By inhibiting TGF-β1/Smad2/3–induced mesothelial-to-mesenchymal transition through a 7-day oral administration, EW-7197 effectively prevented peritoneal adhesion formation and significantly improved healing outcomes in a rat model^11^.

Excessive production of collagen fibers tends to arrange in an irregular pattern and constitutes another reason for suboptimal wound healing. Using short, pulsed electric fields to ablate injured tissues, irreversible electroporation (IRE) has gained increasing attention in the field of tissue regeneration^12^. A recent study demonstrated a significant reduction of collagen in tissue, ranging from 35 to 50%, and downregulation of TGF-β1 after such electrostimulation^13^. Moreover, IRE is thought to accelerate the apoptosis of fibroblasts when regularly performed at certain time intervals after fibroblasts migrate to the injured site, and this feature is critical to improving wound healing because delayed apoptosis of fibroblasts and fibroblast-like cells can severely impact the healing outcomes^14^. With the hypothesis that the combination of EW-7197 with IRE could effectively improve wound healing, this study aimed to evaluate the safety and efficacy of EW-7197 combined with IRE in a rat excisional wound model.

## Results

### Procedural outcomes

**Figure 1:**
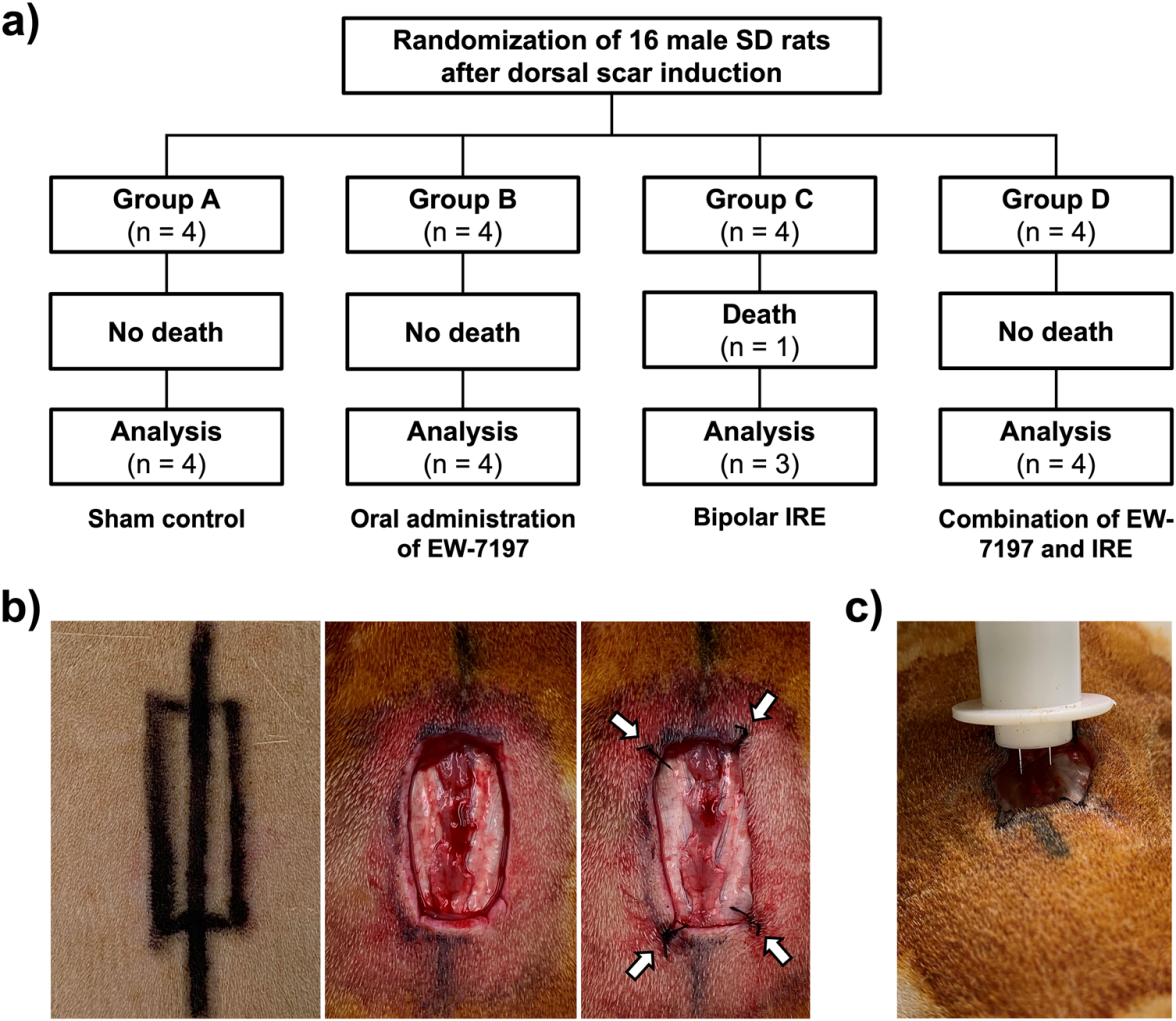
(**a**) Study scheme. (**b**) Technical steps of dorsal excisional wound induction. A 10 × 30 mm^2^ rectangular excision was made to remove the skin, subcutaneous fat, and panniculus carnosus muscle. The skin and panniculus carnosus muscle were then sutured at the four corners (arrows) of the excision to minimize muscle contraction. (**c**) Application of irreversible electroporation. The electrodes were inserted at the lower half of the excisional wound and advanced downward and forward at 45°. *SD* Sprague–Dawley, *IRE* irreversible electroporation.

Dorsal excisional wound was successfully performed in all rats without any procedure-related complications. The oral administration of EW-7197 was successfully administered to all rats in Groups B and D, and IRE was successfully performed on all rats in Groups C and D. One (6.25%) rat from Group C was discovered diseased on day 4 post-procedure and was therefore excluded from further analysis (Fig. 1). While the exact cause of death was uncertain, it was highly likely attributed to the pain induced by the procedure. The remaining 15 (93.75%) rats survived until the end of the study and exhibited scab formation over the wound, which qualified for final analysis.

### Wound closure rate

**Figure 2:**
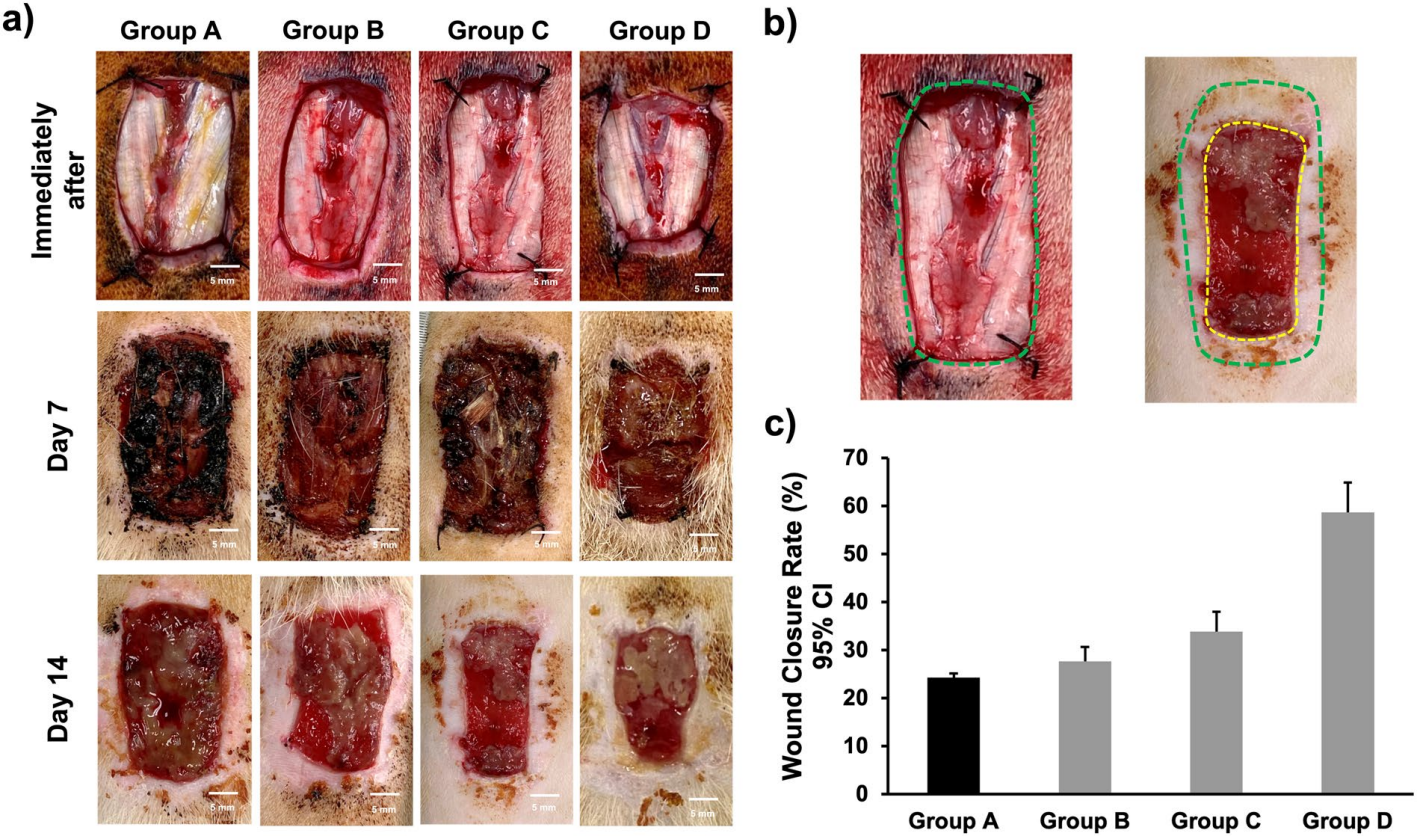
Representative photographs and analysis of the wound closure rates. (**a**) Following the induction of dorsal excisional wounds, scabs formed in all rats during the wound healing process. The wound was smaller in the three treatment groups (Groups B–D) on day 14 after the scab was removed. The wound, after being ablated by irreversible electroporation (IRE; Groups C–D), appeared less dark than those that did not undergo IRE (Groups A–B). (**b**) Representative photograph demonstrating the measurement of wound area; green dotted lines indicate the initial defect and yellow dotted lines indicate the remaining defect. (**c**) Comparison of changes in the wound area among the groups that demonstrated a significant reduction in the wound area after the combination of EW-7197 and IRE was applied. *CI* confidence interval.

Representative photographs of the changes in the wound area are shown in Fig. 2. The mean wound closure rates for Groups A–D were 24.3% ± 0.9%, 27.7% ± 3.0%, 33.8% ± 4.2%, and 58.7% ± 6.3%, respectively. Among all the groups, a statistically significant difference was detected between Groups A and D; B and D; and C and D (*P* = 0.004, 0.006, and 0.012, respectively). No significance was detected between Groups A and B; A and C; and B and C (all *P* > 0.05).

### Laser Doppler scanning

**Figure 3:**
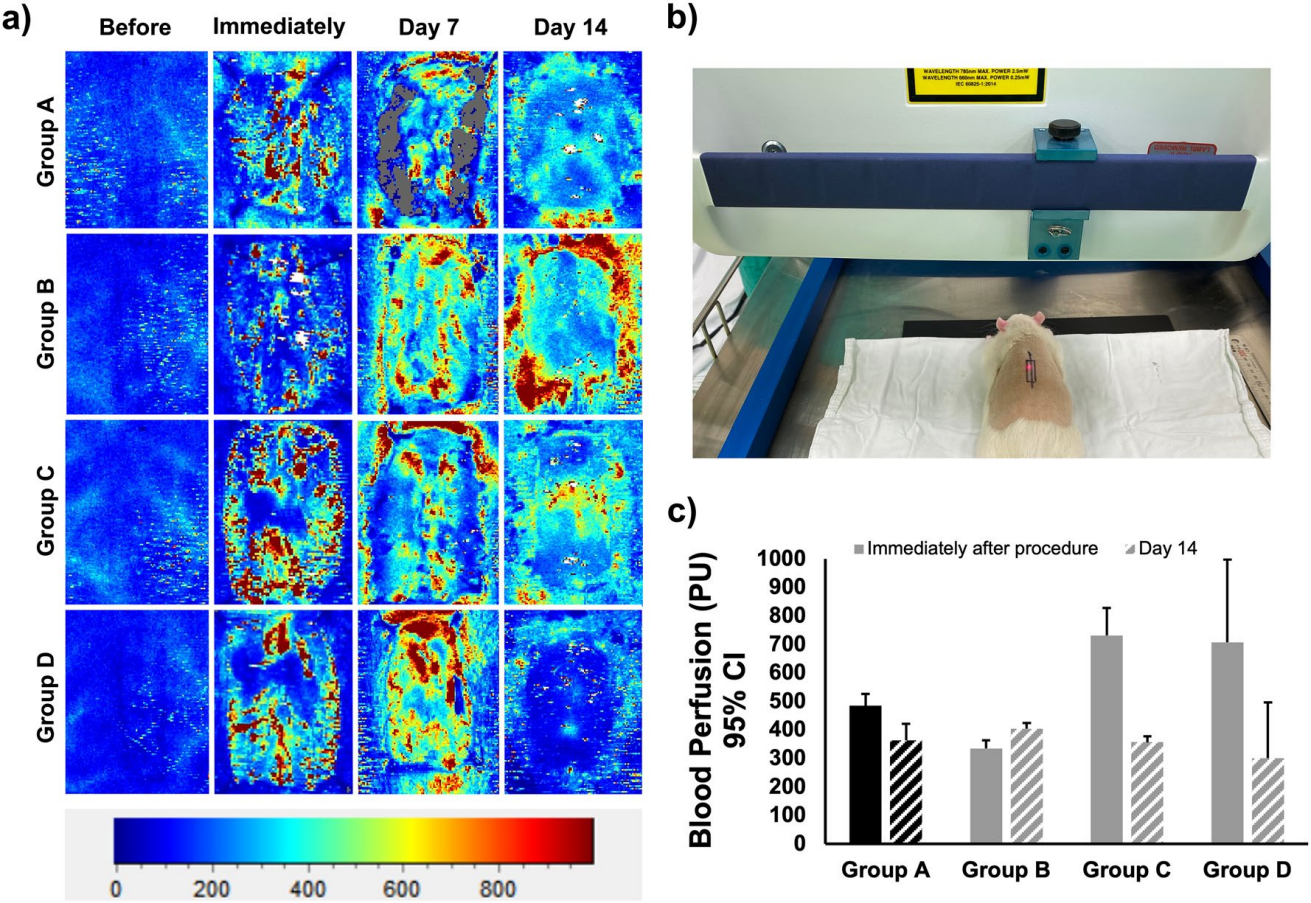
Laser Doppler images and analysis on the change in blood perfusion in the four groups. (**a**) Representative laser Doppler images showing higher blood flow immediately after the procedure. On day 7, increased blood flow was seen in all groups. On day 14, the groups that did not undergo ablation by irreversible electroporation (IRE; groups A–B) demonstrated high blood flow, mostly in the peripheral area, while the high flow was less or not observed in the central area of the wound when IRE was applied (groups C–D). Blue indicates low flow and red indicates high flow. (**b**) Blood perfusion detected by the laser Doppler flowmeter. (**c**) Analysis of the change in blood perfusion showing a significant increase in blood flow immediately after the procedure, especially in the groups receiving IRE (Groups C–D), and blood flow dropped back to a nearly normal level at day 14. *CI* confidence interval.

The results of laser Doppler scanning are shown in Fig. 3. Blood flow significantly increased, especially in the groups involving IRE application (i.e., Groups C and D), immediately and 7 days after the procedure compared to the images taken before the procedure in all rats. On day 14, after removing the scab to allow a thorough view of the wound, the degree of blood flow significantly decreased in all groups to a nearly normal level, which was particularly prominent in Group D, suggesting the most improved healing result for the combination therapy. The blood flow was barely seen in the center of the wound; however, in Group B, the degree of blood flow was higher in the peripheral area than in the other three groups.

### Histological analysis

**Figure 4:**
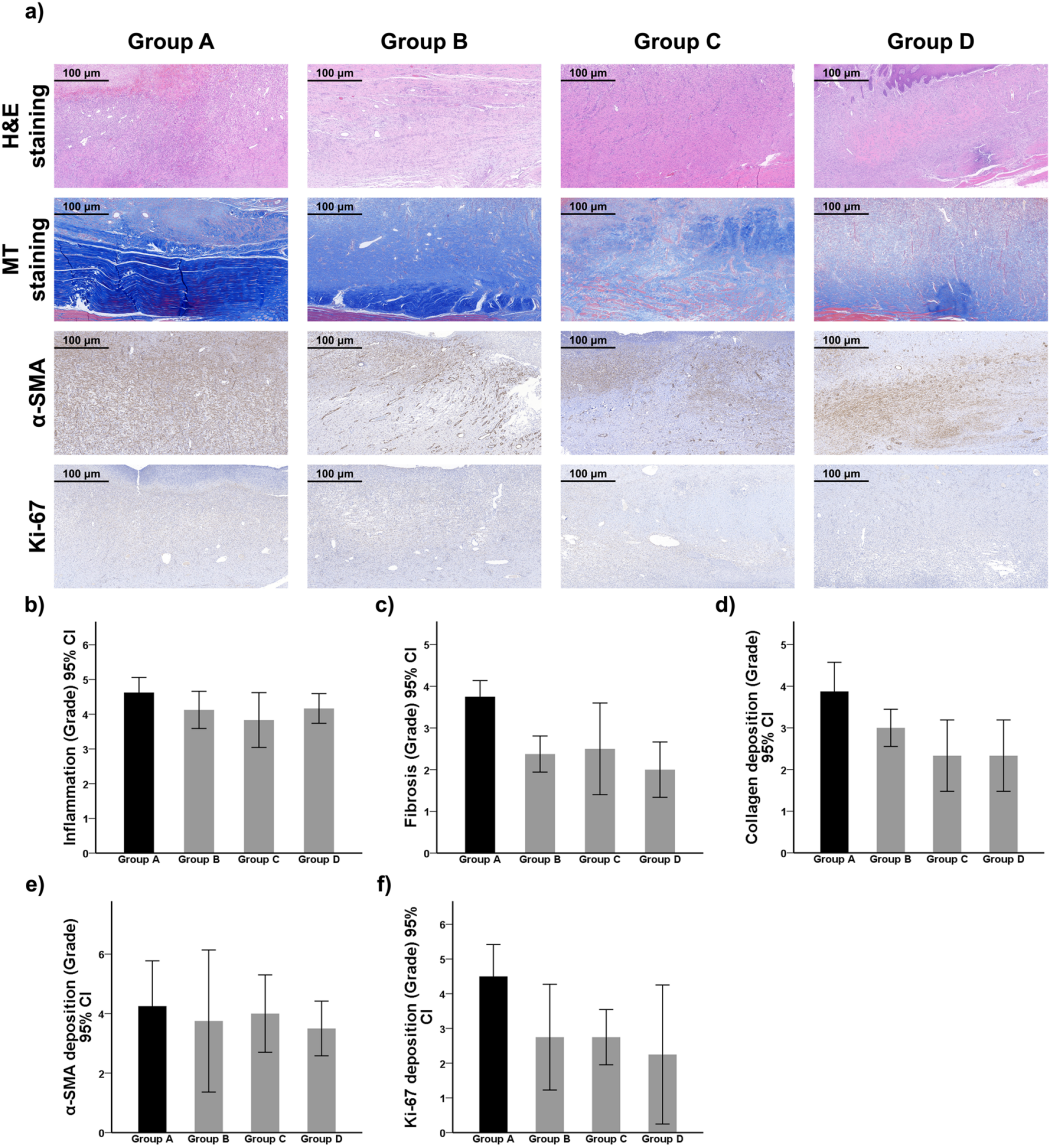
Representative (**a**) microscopic images and (**b**–**f**) histological analysis of the wound. No significant difference was detected in (**b**) inflammation among the groups. All treatments significantly reduced (**c**) the degree of fibrosis compared to Group A. (**d**) The degree of collagen deposition was significantly reduced in Groups C and D compared to Group A. Although a statistically significant difference was not detected in (**e**) the expression of α-SMA–positive cells among the groups, (**f**) the expression of Ki-67–positive cells was significantly inhibited in Group D. H&E hematoxylin and eosin, *MT* Masson’s trichrome, *α-SMA* alpha-smooth muscle actin, *CI* confidence interval.

The summary of histological analyses is presented in Table 1, and representative histological findings are presented in Fig. 4. No statistically significant difference was found in the mean inflammation scores between the control and treatment groups or among any two treatment groups (all *P* > 0.05), though the score was decreased in all treatment groups. For the mean fibrosis score, Group D generated the lowest score followed by Group B where EW-7197 was orally administered for 7 days (Groups A vs. D, *P* < 0.001; Groups A vs. B, *P* = 0.002). Applying only IRE to the wound also lowered the mean fibrosis score to a significant level when compared to that of the control group (Groups A vs. C, *P* = 0.011). However, a statistically significant difference could not be generated between any two treatment groups (all *P* > 0.05). Although the mean collagen deposition score was lower in all treatment groups, a significant difference was only detected between Groups A and C and between Groups A and D in which IRE was involved (both *P* = 0.005).

**Table 1:** Comparison of histological and immunohistochemical findings.

	Group A	Group B	Group C	Group D		
Inflammation (grade)	4.63 ± 0.52	4.13 ± 0.64	3.83 ± 0.75	4.17 ± 0.41		
Fibrosis (grade)	3.75 ± 0.46	2.38 ± 0.52	2.50 ± 1.05	2.00 ± 0.63		
Collagen deposition (grade)	3.88 ± 0.83	3.00 ± 0.53	2.33 ± 0.82	2.33 ± 0.82		
α-SMA (grade)	4.25 ± 0.96	3.75 ± 1.50	4.00 ± 0.82	3.50 ± 0.58		
Ki-67 (grade)	4.50 ± 0.58	2.75 ± 0.96	2.75 ± 0.50	2.25 ± 1.26		

Groups	A vs. B	A vs. C	A vs. D	B vs. C	B vs. D	C vs. D
Statistical analysis						
Inflammation	0.351	0.090	0.492	0.799	0.999	0.799
Fibrosis	0.002	0.011	< 0.001	0.986	0.733	0.580
Collagen	0.119	0.005	0.005	0.375	0.375	> 0.999
α-SMA	0.898	0.985	0.731	0.985	0.985	0.898
Ki-67	0.065	0.065	0.016	> 0.999	0.851	0.851

Note: All data are presented as mean ± standard deviation.

### Immunohistochemical findings

As shown in Table 1 and Fig. 4, no statistically significant difference was observed in the expression of α-smooth muscle actin (α-SMA)–positive cells between the control and treatment groups, nor among any two treatment groups, but the score was lowered in all treatment groups. In terms of the expression of Ki-67–positive cells, except for Group D where the expression was significantly lowered after the combination therapy was given (Groups A vs. D, *P* = 0.016), it was lowered in number in Groups B and C when compared to the control group but at a non-significant level (Groups A vs. B and Groups A vs. C, both *P* > 0.05). Furthermore, a significant difference was not produced between any two treatment groups (all *P* > 0.05).

## Discussion

This study evaluated the safety and efficacy of the combination of EW-7197 with IRE for improving wound healing in a rat excisional wound model. Although the monotherapy of EW-7197 and IRE reduced the degree of inflammation, α-SMA expression, and Ki-67 expression in numbers, the comparison with the control group did not show statistical significance. Except for Groups C and D, which had a significantly lower degree of collagen deposition compared to that in Group A (*P* = 0.005), the difference between any two other groups showed no statistical significance. In contrast, the fibrosis score was significantly reduced in all treatment groups, and the change was most prominent in Group D where both EW-7197 and IRE were administered. The expression of collagen deposition and Ki-67 was also significantly lower in Group D than in Group A. In addition, Group D generated the highest wound closure rate (58.7% ± 6.3%), which was comparable to a previous study (57.9%). The laser Doppler scanning results also demonstrated an obviously higher degree of blood flow immediately after wound induction in Group D, suggesting a higher degree of angiogenesis, an essential process for reversing hypoxia for appropriate and durable tissue repair, was induced during the early phases of wound healing by IRE^15^. Taken together, the healing outcome can be concluded as the most effective when both EW-7197 and IRE were administered by suppressing the overexpression of fibrosis and reducing excessive collagen deposition simultaneously.

Wound healing is a complex process and consists of three distinct but overlapping phases, which are inflammation, proliferation, and remodeling^16^. A possible underlying mechanism occurred in this current study is: (1) electrostimulation immediately after wound induction significantly reduced collagen production in the wound, considering collagen’s role in attracting fibroblasts to the injured site, further differentiating into myofibroblasts and causing overproduction of ECM^13,17^; and (2) EW-7197 administration for 7 days after wound induction inhibits the TGF-β signaling pathway, whose persistent activation enables myofibroblasts to continuously produce ECM, leading to compromised wound healing and pathological scarring^18^. Initial inflammation is helpful for wound re-epithelization and closure by activating multiple cell populations in the dermis and epidermis; however, prolonged and increased inflammation leads to suboptimal wound healing and subsequent fibrotic conditions^19^. As inflammasome activation upregulates the production of pro-inflammatory cytokines, including TGF-β, administering its inhibitor (i.e., EW-7197 in this case) can effectively block this signaling pathway and thus minimize the consequences of detrimental scarring. After injuries occur, immediate actions (e.g., early specifically targeted treatment) need to be taken to halt the disease process^20^. Detrimental scarring, one of the most severe consequences, has been a clinical challenge for a long time, and the existing treatment options may be deficient. For instance, the conventional approach of parenteral steroid treatment is also associated with a higher risk of inducing lower bone density, resulting in osteoporosis and osteopenia^21^. Therefore, with the anticipation of improving the treatment outcome without causing significant patient discomfort and severe adverse effects, there is an urgent need to explore more promising treatments. Electrochemotherapy is a well-established technique for the treatment of benign or malignant lesions including dermal lesions^22^. With the increasing availability of electroporation, recent investigations on curing scars rely on electrochemotherapy, which combines electroporation and a pharmacologic agent, such as bleomycin and calcium^23^. Electrochemotherapy in combination with various chemotherapeutic agents might represent a trending treatment strategy for improving wound healing and reducing scar formation. Two common approaches for administering the chemotherapeutic agent are intralesional injection and oral administration^24^. Unlike previously investigated agents that were directly injected into the lesion, EW-7197 is a potent oral TGF-β1 receptor kinase inhibitor that inhibits the signaling pathway of TGF-β/Smad and abrogates TGF-β1–induced cell migration and invasion. In clinical settings, the advantages of oral drugs include reduced pain and high patient adherence due to their non-invasive nature and convenience^25^. Moreover, continuous oral drug administration might create a more lasting treatment effect than a one-time injection.

An important feature of electroporation is its various types of parameters and components, such as the shape of the electrode (e.g., needle electrode and plate electrode), voltage, and application frequency. However, given the limited applications in promoting wound healing, the agreement on the optimal IRE parameters may not have been achieved. Taking Manca et al*.*’s study as an example, the selection between plate or needle electrodes was made according to the lesion size or thickness, but a detailed selection criterion remains unclear^26^. Although previous studies suggested repeating IRE every 10–20 days, this frequency could not be adopted considering the needle electrodes available for this study to prevent unnecessary multiple injuries to the wound site. Given the wide range of IRE parameters, this present study did not focus on testing different parameters but rather assessed whether treating in combination with IRE would improve the treatment outcomes. The effects produced by different combinations are worth further investigation in subsequent trials.

This study has some limitations. First, the sample size was relatively small to generate a more robust analysis but met the ethics requirement for animal experiments, and the follow-up period was short. Subsequent studies in animals, and more preferably in phase I clinical trials, with larger sample sizes and longer study periods are required to further verify these findings. Second, due to the inherent limitation of an animal study, only the morphology of the wound can be assessed, while other symptom relief, such as pruritus and itching, can hardly be evaluated^26^. However, previous clinical trials claimed that pruritis usually got ameliorated as the wound became smaller, despite the possibility of being influenced by a placebo effect^21^. Fourth, the scab formed on all rats and affected some measurements (e.g., laser Doppler scanning) throughout the follow-up period. It was not removed during follow-up because the removal may induce a new wound and consequently influence the analysis. Lastly, since plate electrodes were not available for this study, needle electrodes were selected instead, and the procedure may be less tolerable to the animals than a non-invasive treatment. However, the change in blood flow was believed to be sufficiently obvious even without being quantified. To further enhance treatment outcomes, plate electrodes are better used in the above-mentioned subsequent studies to minimize invasiveness to the animals. Also, for trials at a clinical level, plate electrodes are required to avoid pain and further trauma to the wound site in patients suffering suboptimal healing outcomes.

In conclusion, the combination of EW-7197 administration and IRE may represent an effective treatment for improving wound healing in a rat excisional wound model. Through inhibiting TGF-β levels by orally administering EW-7197 for consecutive 7 days and suppressing excessive collagen formation by IRE, the group receiving combination therapy demonstrated the highest wound closure rate, the lowest fibrosis expression, and a nearly normal blood perfusion level at 14-day follow-up. Furthermore, although the IRE procedure in this present study was invasive to the animals due to the unavailability of appropriate electrodes, the procedure can be rendered non-invasive to be safely performed on a regular basis, ensuring a more tolerable procedure while further enhancing the healing outcomes. This combination therapy may be introduced as a promising new treatment option for improving wound healing during the early phases of the process.

## Methods

### Animal study design

This study was approved by the Institutional Animal Care and Use Committee of Asan Medical Center (IACUC-13-315) and conformed to the principles outlined in the *National Institutes of Health Guide for the Care and Use of Laboratory Animals*. Also, this study was conducted in compliance with the *ARRIVE guidelines*. Sixteen 12-week-old male Sprague–Dawley rats (mean weight, 396.91 g; JA BIO, Suwon, Korea) were used. Following dorsal excisional wound induction, the rats were randomly divided into four groups, consisting of four rats in each. The groups were as follows: Group A (sham control, receiving no treatment), Group B (oral administration of EW-7197 for 7 days), Group C (one-time application of IRE), and Group D (one-time application of IRE followed by oral administration of EW-7197 for 7 days) **(**Fig. 1a). IRE was performed immediately after dorsal excisional wound induction on the study day, and EW-7197 was orally administered starting the day after wound induction. All animals were individually housed, maintained on a 12 h light/dark cycle, kept at controlled environmental temperature (24 ± 1 °C) and humidity (55% ± 10%), and provided with unrestricted access to food and water. After 14 days from the procedure, all rats were euthanized using inhalable carbon dioxide for further exploration.

### Dorsal excisional wound induction

To create the dorsal excisional wound model, all rats were initially anesthetized by intramuscular administration of 50 mg/kg zolazepam and tiletamine (Zoletil 50; Virbac, Carros, France) and 10 mg/kg xylazine (Rompun; Bayer Healthcare, Leverkusen, Germany). Dorsal hair was removed using an electric clipper, and a 10 × 30 mm^2^ rectangular excision was performed to remove the skin, subcutaneous fat, and panniculus carnosus muscle^27^. Subsequently, the skin and panniculus carnosus muscle were sutured at the four corners of the excision to minimize muscle contraction, which could interrupt the evaluation of wound healing potential (Fig. 1b)^28^. For postoperative pain control, 0.05 mg/kg ketorolac tromethamine (Keromin, Hana Pharmaceutical, Seoul, Korea) was intramuscularly administered for 3 days. Antibiotics were not administered to avoid systemic or local inflammation reduction^29^.

### EW-7197 administration

EW-7197 was provided by the EWHA DrugDesignHouse, Co., Ltd. (Seoul, Korea), and the dosage of 20 mg/kg/d was determined based on previous studies^11,30^. Daily gastric gavage was delivered through flexible feeding tubes (Fuchigami, Kyoto, Japan) to orally administer EW-7197 suspended in 1 mL of vehicle (ddH_2_O) for 7 days starting the day after dorsal excisional wound induction.

### IRE procedure

The protocol used in this study included two essential components: application of an electric field strength corresponding to 125 V/mm and delivery of 200 pulses^31^. The needle electrodes (BTX Genetronics, San Diego, CA, USA) were spaced at 5-mm intervals and inserted into the lower half of the wound, then advanced downward and forward at a 45° angle (Fig. 1c).

### Measurement of wound closure rate

A gross examination of the wound was performed immediately, 7, and 14 days after the procedure. Corresponding photographs were taken at each time point and were used to measure the wound area using the ImageJ software (version 1.53k; NIH, Bethesda, MD, USA) after removing the scab (Fig. 2b). The wound closure rate was calculated using the following formula^32^:

$$Wound\,closure\,rate = 100\% \times \frac{initial\,defect - remaining\,\,defect}{{initial\,\,defect}}$$

### Laser Doppler scanning

To assess the changes in blood flow of the entire excisional wound during the healing process, a laser Doppler scanner (moorLDI2-HIR, Moor Instruments, DE, USA) was used (Fig. 3b). Measurements were taken before the procedure and immediately, 7, and 14 days after the procedure. The imaging was performed at a distance of 10 cm from the excisional wound. The laser beam (780 nm) detected and processed the reflection from circulating red blood cells in capillaries, arterioles, and venules to produce a computerized, color-coded image. Different colors on the image depict varying degrees of flow (i.e., blue indicated low flow and red indicated high flow), which reflected different levels of perfusion^33^. Image analysis was performed using the Moor LDI2-BI V2 software (Moor Instruments, Axmoor, UK). Mean flux values, representing blood flow, were calculated from the relative flux units for the areas corresponding to the dorsum of the rats and recorded in blood perfusion units^34^.

### Histological examination

Following gross examination, the wounded tissues were harvested for subsequent analyses. The harvested tissues were fixed in formalin for 24 h and embedded in paraffin. Subsequently, the samples were longitudinally sectioned into 4-μm-thick slices and stained with hematoxylin and eosin (H&E) and Masson’s trichrome (MT). Histologic analysis was performed using a digital slide scanner (Panoramic 250 FLASH III; 3D Histech Ltd., Budapest, Hungary), and measurements were obtained with a digital microscope viewer (CaseViewer; 3D Histech Ltd.). The degree of inflammation and fibrosis in the wounded tissues was graded based on H&E staining, and the degree of collagen deposition was graded on MT-stained sections^35^. The level of inflammation, fibrosis, and collagen deposition was graded using a 5-point scale as follows: 1 = mild; 2 = mild-to-moderate; 3 = moderate; 4 = moderate-to-severe; and 5 = severe^30^.

### Immunohistochemistry

Formalin-fixed, paraffin-embedded sections of the wounded tissues were immunohistochemically analyzed following a previously published protocol^36^. The sections were routinely deparaffinized and hydrated before being incubated overnight with primary antibodies specific for Ki-67 (bs-2130R; Bioss, Woburn, MA, USA) and α-SMA (LS Bio, Seattle, WA, USA) markers at 4 °C. Subsequently, the sections were counterstained with H&E and were visualized using the BenchMark XT IHC automated immunohistochemical stainer (Ventana Medical Systems, Tucson, AZ, USA). The degrees of α-SMA– and Ki-67–positive deposition were subjectively determined according to the distribution and density of the cells and graded on a 5-point as follows: 1 = mild; 2 = mild-to-moderate; 3 = moderate; 4 = moderate-to-severe; and 5 = severe.

### Statistical analysis

Data are expressed as mean ± standard deviation. One-way analysis of variance with Tukey’s post hoc test was used to compare the continuous variables. *P* < 0.05 was considered statistically significant. All statistical analyses were performed using the SPSS software (version 27; IBM, Chicago, IL, USA).

## Data Availability

The data that support the findings of this study are available from the corresponding authors upon reasonable request.
